# Preventing parastomal hernias with systematic intraperitoneal specifically designed mesh

**DOI:** 10.1186/s12893-017-0237-7

**Published:** 2017-04-19

**Authors:** Raquel Conde-Muíño, José-Luis Díez, Alberto Martínez, Francisco Huertas, Inmaculada Segura, Pablo Palma

**Affiliations:** 1 Division of Colon & Rectal Surgery, Complejo Hospitalario Universitario de Granada, Granada, Spain; 2 Department of Radiology, Complejo Hospitalario Universitario de Granada, Granada, Spain

## Abstract

**Background:**

Parastomal hernia is a very common complication after stoma formation. Current surgical techniques for repairing parastomal hernia have unsatisfactory results. We aim to assess our preliminary experience with prophylactic mesh placement at the time of stoma formation.

**Methods:**

Data were prospectively recorded. A specifically designed mesh made of polyvinyl fluoride with central conduit (*Dynamesh* IPST®) was fixed using an intra-peritoneal onlay technique. Safety was evaluated by means of surgical data and frequency of mesh-related complications, efficacy by the rate of parastomal hernias.

**Results:**

Thirty-four patients were included in the study. Three of them died before a year of follow up (not related to the stoma), so they were excluded. The other 31 patients (11 women and 20 men) were prospectively followed up after different pathologies resulting in a permanent colostomy. Twelve months after surgery CT-Scan imaging revealed two (6.4%) parastomal hernias, one of them already clinically suspected. During the follow up, 29% of the patients (*n* = 9) developed another type of hernia (incisional, inguinal or both). In five patients (16.1%) a light stomal retraction of the otherwise slightly prominent ostomy was observed. Median clinical follow-up was 17.5 months (range 12–34).

**Conclusion:**

Prophylactic parastomal mesh placement might be a safe and effective procedure with a potential to reduce the risk of parastomal hernia. Routine use of this technique should be further analysed.

## Background

The original reports of stomas were described after abdominal war injuries or from spontaneous fistula development in patients with incarcerated hernia. The pioneer to carry out a colostomy to treat a patient with imperforate anus was Littré in 1710 [1].

Nowadays, ostomy construction is a widespread technique in every surgical department worldwide with a prevalence of up to 700,000 citizens in Europe undergoing ostomy surgery; Further, up to 60% of individuals with an ostomy will never undergo a reversal operation [2, 3]. Unluckily, stomas complications, such us dehydration from high output ostomies, mechanical ileus, skin irritation, prolapse, and herniation, are pretty common [4, 5].

A parastomal hernia (PSH) is a kind of incisional hernia in relation to a formerly constructed stoma. PSH remains a widespread complication amongst citizens with an ostomy, with reported incidences up to 56%, depending on the type of stoma (ileostomy versus colostomy) and the duration and quality of follow-up [6, 7]. Although many PSH stay asymptomatic, they can be a substantial origin of morbidity, with up to a third going for further surgery due to complications [8, 9].

Once a PSH developed, it may be repaired by an open or a laparoscopic surgical approach. Conventional or open techniques include direct repair of the abdominal wall deficiency, opposite relocation of the stoma or prosthetic mesh reinforcement. So far, results obtained from these techniques have been unsatisfactory, with reported recurrence rates of 30 to 76% [10]. The high prevalence of PSH and the technical hitches found during repair surgery mean that it is in the best interest to prevent its occurrence.

In recent times, mesh reinforcement at the time of stoma formation has been advocated to decrease the incidence of PSH [11–15].

The availability of a specially designed mesh made with a central hole and a funnel arising, which can be used intraperitoneally with direct contact to the bowel, was the prerequisite for a preliminary interventional study on prophylactic use of a mesh at the time of stoma formation to evaluate the safety and efficacy in PSH prevention.

## Methods

All elective patients between December 2012 and March 2015 who needed a permanent ostomy were enrolled and followed up prospectively. Patients were offered the mesh implantation on clinical basis as being at particularly high risk for PSH. All patients gave informed consent and understood that this was a new surgical variation of an established technique due to the specifically designed mesh used. The study was reviewed and approved by the Ethics Committee of the University Hospital in Granada. Major complications during the primary procedure were the only exclusion criteria.

### Surgical technique

**Fig. 1:**
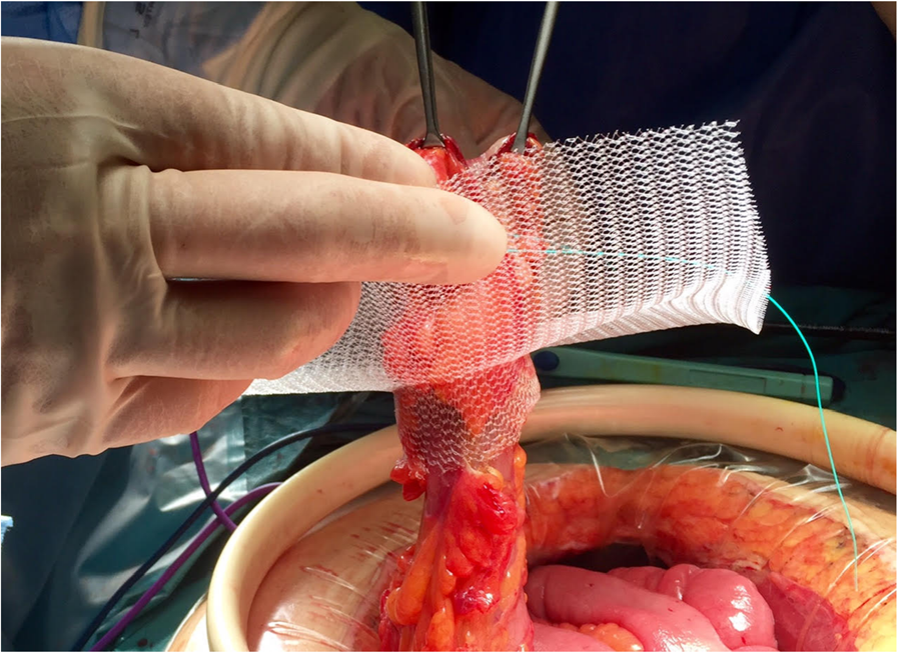
The funnel is oriented to the abdominal cavity and must fit tightly around the bowel, previously closed with a stapling device

**Fig. 2:**
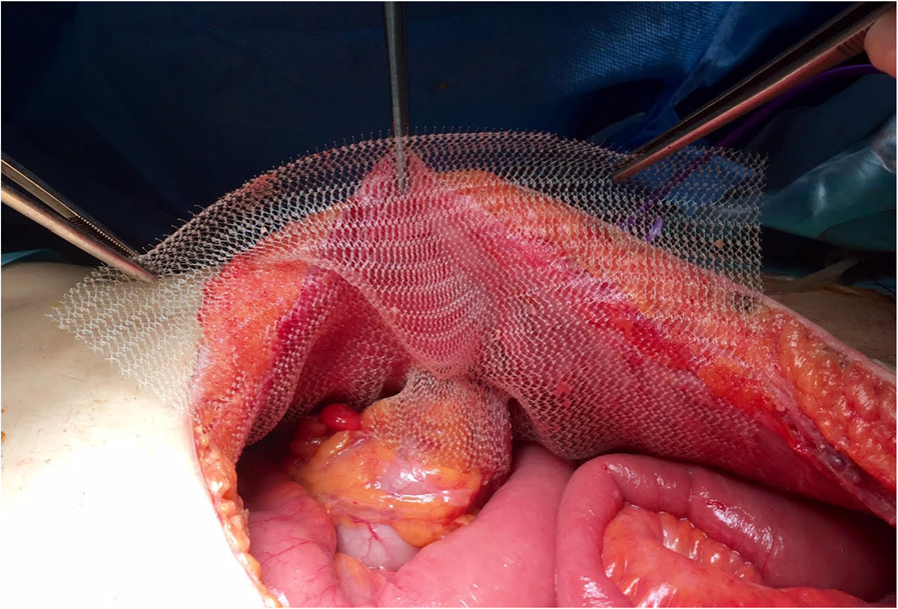
Final intraperitoneal mesh position. The bowel pulled through the funnel is not fixed at the mesh

According to our standardised technique, the proposed bowel for the colostomy is closed with a surgical stapler lineal device, in order to decrease the possibility of contamination. Stoma site was preoperatively marked. The trephine is shaped by oval excision of the skin without any excision of the subcutaneous fat tissue. A cross-shaped incision is made in the fascia, after exposing the rectus sheath and finallythe rectus abdominus muscle is opening in the direction of the fibers. Dynamesh IPST® 16×16 cm. (FEG-Textiltechnik, Aachen, Germany), was used in all cases. Dynamesh is a real mesh structure warp-knitted by polyvinylidene xuoride (PVDF) with a small amount of polypropylene on the parietal side [16, 17]. The bowel is pulled through the central funnel Fig. 1. The funnel, with a length of 3 cm and a diameter of 2 cm. is oriented to the abdominal cavity and must fit firmly around the bowel. The mesh is fixed by single stitches at the four corner-edges Fig. 2.

Safety was assessed by means of perioperative complications and efficacy through the follow up. Postoperative complications, such as skin infections, degree of stomal ingrowths’, prolapse; leakage, necrosis and stenosis are recorded.

**Fig. 3:**
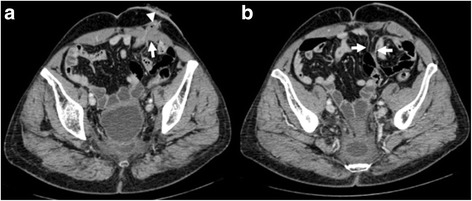
Axial abdominal CT images with a post-contrast portal phase. **a** Normal appearance of colostomy (*arrowhead*) with the adjacent mesh partially observed. In a previous slice **b** it is appreciated the funnel of the mesh oriented to the abdominal cavity

**Fig. 4:**
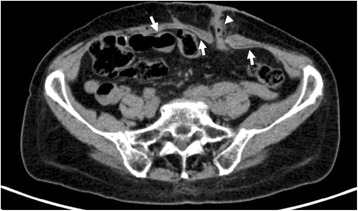
Axial abdominal CT image without intravenous contrast. It can be appreciated the location of the mesh (*arrows*) fitted around the bowel (*arrowhead*)

PSH is defined as any noticeable bulge, in the vicinity of the ostomy with the patient erect, supine, and performing the Valsalva maneuver. Furthermore a CT-scan was performed in the supine position, 12 months after implantation in every case in order to rule out subclinical herniation, Figs. 3 and 4. At CT-scan, parastomal hernia was defined as any intraabdominal content protruding beyond the peritoneum or the presence of a hernia sac Outpatient follow-up is scheduled at 3 weeks, 3 months, 6 months and 1 year postoperatively. In order to avoid inter-observer variability, clinical examination and CT were both performed by a single experienced surgeon and radiologist respectively. All the authors confirm no competing interest.

## Results

Thirty-four patients were included in the study. Three of them died before a year of follow up (not related to the stoma), so they were excluded. The other 31 patients (11 women and 20 men) were prospectively followed after different pathologies resulting in a permanent terminal colostomy/ileostomy. Surgical treatment and underlying diseases are summarized in Table 1.

**Table 1:** Patients characteristics

Patients	N (%)/Median [Range]
Age	63 (41–91)
Obesity (BMI > 30)	
Yes	7 (22.6%)
No	24 (77.4%)
Respiratory disease	
Yes	2 (6.5%)
No	29 (93.5%)
Diagnosis	
Neoplasm	24 (77.4%)
Anastomotic leakage	2 (6.5%)
Other	5 (16.1%)
Diabetes	
Yes	4 (12.9%)
No	27 (87.1%)
Previous hernioplasty	
Yes	2 (6.5%)
No	29 (93.5%)
Surgical techniques	
APR or LAR	27 (87.1%)
Colostomy	3 (9.7%)
Ileostomy	1 (3.2%)

*APR* abdomino-perineal resection, *LAR* low anterior resection with colostomy

Operating time was not lengthened by the implantation of the mesh. Time required for this technique is less than 5 min. Neither related intraoperative adverse effects nor mortality was observed. No ostomy site infections with need of mesh retrieval, leakage, prolapse, necrosis or stenosis were observed. Furthermore, only one PSH could be detected clinically up to that time. Twelve months after surgery CT imaging revealed one further case of PSH. Both together represents a 6,4% PSH rate in our series.

The first patient was a morbid obese woman (BMI 40), with diabetes. An abdominal-perineal excision was performed due to rectal cancer. She developed during the follow up both a parastomal and incisional herniation, both clinically relevant. The other patient was operated because of an anastomotic leakage with pelvic peritonitis after low anterior resection due to rectal cancer; mesh was placed by the 2nd look and Hartmann procedure. Besides a BMI of 28 (overweight), he had no other risk factors and PSH was only assessed upon CT imaging.

During the follow up, 29% of patients (*n* = 9) developed another hernia (incisional, inguinal or both). Three patients died after 12 months of follow up (not related to the stoma), none of them developed PSH. In further five patients (16.1%) a light stomal ingrowth or retraction of the otherwise slightly prominent ostomy was observed, none of the patients required further surgical treatment and were well managed with convexed stoma adhesive skin plate after instruction in the outpatient ostomy clinic. Median clinical follow-up was 17.5 months [range 12–34]. Table 2.

**Table 2:** Results

Results	N (%)
Retraction (stomal ingrowth)	5 (16.1%)
PSH (on clinical examination)	1 (3.2%)
PSH (on CT imaging)	2 (6.5%)
Other hernias during follow up	9 (29%)
Incisional hernia	5 (16%)
Inguinal	2 (6.5%)
Incisional and inguinal	2 (6.5%)
Exitus	3 (9.7%)

## Discussion

Our results of only 6,4% of PSH after mesh prevention, are in accordance with the literature. Further, no mesh-related complications such as stoma stenosis or prolapse, were encountered and none of the implants had to be removed. On the contrary we found six patients with some kind of stomal ingrowth (retraction) demanding convexes stoma devices. Although only 31 patients were finally included and our follow up was 17.5 months, both facts are comparable with the data of already existing publications [14, 15].

Due to the high frequency of PSH and high rate failure of repair techniques, interest has been focused on prevention rather than correction. In fact, the true incidence is underestimated because many PSH remain asymptomatic. Cingi et al. showed that 52% of their patients with a colostomy had a parastomal hernia at clinical examination, while additional computed tomography yielded an incidence of 78% [18]. For this reason, a CT was performed in all our patients 12 months after surgery, which is the minimum length of follow-up to assess the development of PSH [19], and indeed the second PSH detected in our study was appraised upon imaging with no clinical complaint.

Prophylactic mesh placement at the time of primary stoma formation is the only efficient way to achieve this goal and appears to be a cost-effective strategy in patients after abdomino-perineal excision in rectal cancer [20]. This idea was first implemented by Bayer et al. in 1979 in which they reinforced the stoma site with Marlex mesh in 43 patients [21]. For pathogenetic reasons, a mesh-based reinforcement seems to be inevitable to avoid a PSH, in fact increasing evidence of impaired wound healing in incisional hernia supports routine use of a mesh repair [22].

A pioneer randomized study showed the impressive decline of PSH formation after retromuscular positioning of an incised mesh [13]. Since that time, further prospective studies appeared, also indicating a variable incidence of PSH or even other stoma complications linked with the procedure, depending mostly on the type of mesh, its anatomical positioning and the technique used [14, 15, 23–26].

A systematic review in 2012 including three randomized controlled trials and a total of 128 patients, confirmed a important difference in the incidence of PSH between controls and patients with prophylactic mesh [27]. The incidence was 12.5% for those with mesh and 53% for controls with no difference in mesh-related morbidity. Further studies using a synthetic prosthesis with a minimum follow-up of 1 year have shown contradictory results, varying from effective prevention of PSH [28, 29] to unexpected misfortunate outcomes with a high frequency of PSH in the mesh group assessed both clinical and radiologically [26, 30, 31].

The literature shows that both patient and operative technical issues have been mixed up in the subsequent risk of PSH. In fact, individual patient characteristics that have been revealed to be independent risk factors for PSH development include older age, obesity (BMI > 30), respiratory disease, neoplasm, and diabetes mellitus [4, 32, 33]. These factors should theoretically also be a risk for PSH after the prophylactic use of a mesh; Table 1 shows our patients characteristics including all factors mentioned, although none of them influence the prophylactic efficacy of our mesh, both patients with PSH were besides obese and operated because of malignancy, diabetic (one of them) and showed peritonitis after anastomotic leakage (the other one).

Interestingly, records from different studies have varied as to whether the mesh was placed in different anatomical sites. To date, there is no strong evidence to support one method over the other [15, 24, 30, 34–36]. In fact, the common denominator of all these reports was to employ a similar surgical technique. A mesh with a central aperture placed in onlay [24], sublay [12, 36], inlay [37] or intra-peritoneal position was used [30]. Using this technique enlargement of the orifice created in the center of the mesh and augmentation of the extent of the abdominal fascia aperture favoring development of PSH was reported [29]. This technique was described as the “keyhole technique” as opposed to the “modified Sugarbaker technique” [14, 28] in which a non-slit covering mesh is used to correct the PSH.

The technique used in our study differs from the above-mentioned techniques by intraperitoneal placement of a specially designed mesh with a central conduit or funnel directed against the abdominal cavity that should prevent the hernia by overlapping the abdominal wall around the stoma. Further, the bowel pulled through is tightly surrounded by the funnel, thus avoiding possible migration of the mesh and bowel prolapse. In addition, our surgical technique avoids potential surgeon variability by using a preformed mesh, thus making the procedure easier to standardize.

All this factors could explain our PSH rate of only 6,4% after mesh prevention which are in accordance with a recent retrospective multicenter study on 80 patients, using the same mesh and technique as we reported; with a median follow-up of 21 months, PSH developed in three patients (3.75%) and ostomy-related complication in seven (8.75%) but no mesh-related complications were encountered and none of the implants had to be removed [38]. A further advantage of the intraperitoneal onlay mesh technique is that time demand for mesh implantation is reduced using this technique compared with a retromuscular or prefascial location were extensive dissection is required.

Although the possibility of an infection when the bowel, which is contaminated at the stapled end, is pulled through and comes into contact with the mesh, always exist, no infections were observed in our study. The lack of any mesh related infectious complications is also in accordance with previously published studies [24]. In this context, intraperitoneal mesh placement during laparoscopic incisional hernia repair techniques was shown to be related with a lower infection rate compared with conventionalprocedures, which need a retromuscular or prefascial dissection before mesh fixation [39]. This advantage is probably due to avoiding the dissection of the abdominal wall layers, thus preventing hematomas or seromas. Therefore, the intraperitoneal onlay mesh position may be not only less technical and time demanding, but also more tissue friendly.

A prerequisite for that purpose is a material which allows direct contact with the viscera. Dynamesh IPST® is a 3D inversely funnel shaped mesh structure made of PVDF, which is an inert material and has been shown not to induce adhesions to the bowel [16]. Furthermore, it has been experimentally tested that the mesh is well incorporated and effectively prevents adhesions to intestinal structures [17]. The mesh has some elasticity in both directions, comparable with the human abdominal wall [16]. The small amount of polypropylene on the parietal side provides strong mesh incorporation and fixation.

Other complications, such as stoma stenosis or prolapse, have not been observed in our study. On the contrary we found 6 patients with some kind of stomal ingrowth (retraction) demanding convexes stoma devices. Longer follow up should clarify any fibrosis, stenosis or erosion of the bowel-mesh funnel interaction not observed in our study.

Although PSH could occur within 2 years after creation [33, 40], we performed a CT scan in all our patients 12 months after implantation. Further, in order to avoid inter-observer variability, clinical examination and CT were both performed by a single experienced surgeon and radiologist respectively. Both facts led us to feel quite confident about the efficacy in PSH prevention with systematic intraperitoneal prosthetic mesh.

## Conclusion

Prophylactic use of an intraperitoneal onlay *Dynamesh* IPST® to reinforce the abdominal wall and prevent PSH showed in our study to be safe and effective. But for two cases detected (6,4%), no PSH or stoma-related complications were noticed in our series, after a median clinical follow-up of 17.5 months [range 12–34], including CT-imaging after 12 months. Furthermore, the procedure is neither time nor technical demanding. Our data justifies larger clinical randomised trials to prove the regular indication of this specifically designed mesh for every permanent stoma.

## Abbreviations

BMI: Body mass index
CT-scan: Computed tomography scan
PSH: Parastomal hernia
PVDF: Polyvinylidene Xuoride
